# Adiponectin levels in the serum and cerebrospinal fluid of amyotrophic lateral sclerosis patients: possible influence on neuroinflammation?

**DOI:** 10.1186/s12974-017-0861-2

**Published:** 2017-04-20

**Authors:** Patrizia Bossolasco, Raffaella Cancello, Alberto Doretti, Claudia Morelli, Vincenzo Silani, Lidia Cova

**Affiliations:** 1 Department of Neurology and Laboratory of Neuroscience, IRCCS Istituto Auixologico Italiano, piazzale Brescia 20, 20149 Milan, Italy; 2 Diabetes Research Laboratory, IRCCS, Istituto Auxologico Italiano, via Ariosto 13, 20145 Milan, Italy; 3 “Dino Ferrari” Centre, Department of Pathophysiology and Transplantation, Università degli Studi di Milano, via Sforza 35, 20122 Milan, Italy; 4 Laboratory of Neuroscience, IRCCS Istituto Auxologico Italiano, via Zucchi 18, 20095 Cusano Milanino, Milan Italy

**Keywords:** Adiponectin, Adipokine, Neurodegeneration, Neuroinflammation, Motor neuron disease, Fronto-temporal dementia

## Abstract

**Background:**

Adiponectin (APN) is a key player in energy homeostasis strictly associated with cerebrovascular and neurodegenerative diseases. Since APN also belongs to anti-inflammatory-acting adipokines and may influence both neuroinflammation and neurodegenerative processes, we decided to study the APN levels in amyotrophic lateral sclerosis (ALS) and other neurodegenerative diseases.

**Methods:**

We assessed APN levels by ELISA immunoassay in both the serum and cerebrospinal fluid of a cohort of familial and sporadic ALS patients, characterized by normal body mass index and absence of dysautonomic symptoms. The screening of serum APN levels was also performed in patients affected by other neurological disorders, including fronto-temporal dementia (FTD) patients. Means were compared using the non-parametric Wilcoxon test, and Pearson’s or Spearman’s rho was used to assess correlations between variables.

**Results:**

In the whole ALS group, serum APN levels were not different when compared to the age- and sex-matched control group (CTR), but a gender-specific analysis enlightened a significant opposite APN trend between ALS males, characterized by lower values (ALS 9.8 ± 5.2 vs. CTR 15 ± 9.7 μg/ml), and ALS females, showing higher amounts (ALS 26.5 ± 11.6 vs. CTR 14.6 ± 5.2 μg/ml). This sex-linked difference was significantly enhanced in familial ALS cases (*p* ≤ 0.01). The APN levels in ALS cerebrospinal fluids were unrelated to serum values and not linked to sex and/or familiarity of the disease. Finally, the screening of serum APN levels in patients affected by other neurological disorders revealed the highest serum values in FTD patients.

**Conclusions:**

Opposite serum APN levels are gender-related in ALS and altered in several neurological disorders, with the highest values in FTD, which shares with ALS several overlapping and neuropathological features. Further investigations are needed to clarify the possible involvement of APN in neuroinflammation and neurodegeneration.

**Graphical Abstract:**

Possible involvement of APN in neuroinflammatory neurodegenerative diseases

## Background

Metabolic dysfunctions characterize neurodegenerative disorders, such as Alzheimer’s disease (AD) and Parkinson’s disease (PD), wherein early abnormal lipogenesis in glial cells appears a possible mediator of neuronal degeneration [1]. Several studies on the involved biological pathways indicate an active role of signalling molecules secreted by adipose cells, named “adipokines”. Adipokines act as hormones modulating a wide range of physiological metabolic responses from hunger/satiety and energy balance to inflammation, cardiovascular function and reproduction, thus connecting adipocytes to all tissues [2]. Adiponectin (APN) is the most abundant adipokine in the body and is a key player in the signalling network of adipose tissue. Different biological cascades (i.e. neurogenesis, inflammatory response, energy balance of skeletal muscle) as well as pathological conditions (i.e. diabetes, hypertension, atherosclerosis and stroke) [3, 4] involve APN action/activation. Accumulating evidence suggests that APN-mediated modulation of macrophage function and phenotype contributes to its role in controlling inflammation. Adiponectin inhibits the transformation of macrophages into foam cells, reduces intracellular cholesteryl ester content in human macrophages by suppressing the expression of class A scavenger receptors and can bind to apoptotic cells and facilitate their uptake by macrophages. Furthermore, APN levels are increased, rather than decreased, in a number of chronic inflammatory and autoimmune diseases [5]. An emerging close association between neurodegenerative diseases and metabolic dysfunctions is suggested by recent literature, linking APN to the central nervous system (CNS) [2]. Subjects suffering from neurodegenerative disorders, such as AD, PD, multiple sclerosis and Huntington’s disease, display altered serum APN levels [2, 3, 6]. Increased levels of APN may be viewed as a rescue mechanism to counteract the ongoing neuronal loss, even if dual and opposite effects of APN on neuroinflammation, oxidative stress and neuronal apoptosis have been reported [2, 3, 7–11]. Hence, in this controversial scenario, the APN impact on different neurological conditions remains undefined, as well as the eventual ability of APN subforms to cross the blood-brain barrier (BBB) [3]. Nevertheless, exogenous brain APN increase is able to directly influence microglia as well as brain macrophage phenotype and activation state, thus reducing neuroinflammation and depressive-like behaviours in mice [8]. Interestingly, higher serum APN values have been associated with neuroimaging markers of neurodegeneration and cognitive impairment among women in the Mayo Clinic Study of Aging [6].

Amyotrophic lateral sclerosis (ALS) is a fatal adult motor neuron disorder characterized by a progressive motor neuron loss. Recent evidence indicate that ALS is a multifactorial disorder rather than a pure neuromuscular disease [12] and several biological processes are involved in its pathogenesis [13], such as protein aggregation, glutamate excitotoxicity and neuroinflammation [14]. The chief mediators of neuroinflammation in ALS are microglial cells along with other participants (astrocytes, oligodendrocytes, T lymphocytes) [15]. Neuroinflammation and neurodegeneration are shared among AD, PD, cerebrovascular disease and ALS, wherein neuroinflammation shifts from a neuroprotective anti-inflammatory role, characterizing early pathological stages, to a diffuse neurotoxic pro-inflammatory state, typical of late phases [15]. The dual action of neuroinflammation along ALS onset and progression may be related to the selective modulation of pro- and anti-inflammatory cytokines, including some adipokines, well-known modulators of energy balance [16]. Interestingly, energy metabolism is impaired in both sporadic (sALS) and familiar ALS (fALS) patients [12, 17]. Hypermetabolism, weight loss, hyperlipidaemia and impaired glucose tolerance have been reported in ALS [17], although they are apparently related to advanced disease severity [18]. In fact, inclusions of TDP-43 (43-kDa trans-activation-responsive region DNA-binding protein) characterize most of ALS brain tissues whereas the naïve protein regulates fat deposition and glucose pathway [12, 19]. At diagnosis, patients usually have a normal or low body mass index (BMI) and body fat amount seems to be inversely related to ALS risk [12]. However, with disease progression, patients usually reduce food intake, partially due to dysphagia progression, with a consequent weight loss, muscle mass reduction and paradoxically increased energy expenditure [12, 17, 20, 21].

Adipose tissue distribution differs between controls and ALS patients wherein a positive correlation has been reported between the metabolic state/body fat percentage and ALS duration/augmented risk of complications [12]. Latest evidence indicate that subcutaneous fat is predictive of better survival in a gender-related manner [12]; besides, increasing body fat may be associated with a decreased risk to develop ALS and to a longer survival [17].

At present, APN modulation and involvement in neuroinflammation and neurodegeneration are poorly investigated in ALS patients. Interestingly, ALS and one of the less common forms of dementia, fronto-temporal dementia (FTD), share common neurological features together with distinct changes in metabolism, as recently reported [13, 22, 23].

The purposes of our study were to assess (1) serum and cerebrospinal fluid (CSF) APN levels in both fALS and sALS patients in comparison to age- and sex-matched normal weight healthy controls, exploring any possible correlation to clinical parameters, and (2) serum APN levels in other neurological diseases, including FTD.

## Methods

### Participants

This study was conducted in accordance with the Declaration of Helsinki. The research protocol was approved by the ethics committee of IRCCS Istituto Auxologico Italiano, and written informed consent was obtained from all participants.

### ALS patients and controls

A total of 88 serum samples were collected: 36 from healthy lean controls (CTR), genetically unrelated to the patients, not suffering from any known disease conditions and specifically not affected by neurological diseases, and not underweight (BMI ≤18.5 kg/m^2^). Controls were compared to 52 clinically diagnosed ALS patients, matched for age, sex and BMI (see details in Tables 1 and 2). All our patients possess normal BMI. The fALS group included patients carrying mutations in *SOD1* (2 males and 3 females), *C9ORF72* (1 male and 3 females) and *TARDBP* (1 male) genes. ALS patients fulfilled both criteria defined by the revised El Escorial criteria (categories: laboratory-supported probable, probable and definite [24, 25]), with the exclusion of patients affected by possible ALS, and the Awaji criteria (categories: probable and definite [26]). Dementia was diagnosed in 4 female ALS patients [27]. Both patients with spinal and bulbar onset and without swallowing impairment or metabolic alterations at the time of sample collection were enrolled. Analyses of CSF were restricted only to ALS patients (23 samples) due to ethical concerns (additional details in Tables 1 and 2). Additionally, 3 FTD CSFs were analysed since available for clinical reasons. All patients were treated with the standard drug for ALS (riluzole, 100 mg daily). Weight and height were measured and BMI (kg/m^2^) calculated for all patients.

**Table 1:** Characteristics of patients with ALS and control subjects (CTR) in the whole cohort and divided by gender

	Whole cohort		Females		Males	
	CTR	ALS	CTR	ALS	CTR	ALS
No. of serum samples	36	52	17	27	19	25
No. of CSF samples		23		12		11
Age (years)	52.8 ± 17.8	57.9 ± 14.1	49.6 ± 19.1	59.1 ± 14.2	55.7 ± 16.6	56.6 ± 14.2
BMI (kg/m^2^)	22.5 ± 2.5	23.6 ± 3.4	22.6 ± 2.5	23.3 ± 2.6	22.4 ± 2.6	23.8 ± 3.8
Fasting glucose (mg/dl)	88.1 ± 10.9	90.9 ± 13.8	85.5 ± 19.1	87.9 ± 12.8	90.2 ± 12.6	93.5 ± 14.4
Total cholesterol (mg/dl)	192.2 ± 25.5	210 ± 44.6	185.4 ± 23	219.3 ± 42.3^b^	199.7 ± 27	201 ± 45.8
HDL (mg/dl)	56.9 ± 17.8	60.4 ± 17.4	60.7 ± 22	69.8 ± 15	53.8 ± 13.2	51.3 ± 14.5^c^
LDL (mg/dl)	117.2 ± 39	137.1 ± 37	107.2 ± 23.3	134.9 ± 37.8	127.2 ± 49.6	138.9 ± 37.4
Triglycerides (mg/dl)	94.2 ± 25.3	103.1 ± 29.6	92.0 ± 31.1	97.9 ± 25.8	96.1 ± 19.5	108.1 ± 32.6
GOT (AST) (U/l)	18.2 ± 5.6	25.6 ± 10.6^a^	17.5 ± 6.2	22.5 ± 8.3	18.9 ± 5.3^b^	28.5 ± 11.9^c,d^
GPT (ALT) (U/l)	18.2 ± 9.3	29.90 ± 16.89^a^	16.6 ± 6.1	24.3 ± 15.5	19.6 ± 11.5^b^	33.1 ± 16.4^c,d^
GTP (γ-GT) (U/l)	17 ± 6.5	25.8 ± 21.3	15.2 ± 5.9	18.4 ± 14.6	18.8 ± 6.8	33.6 ± 24.6 ^c,d^
White blood cells (*n*)	6.9 ± 2.2	6.7 ± 1.7	6.8 ± 1.8	6.3 ± 1.7	7 ± 2.6	7 ± 1.7
Lymphocytes (*n*)	28.9 ± 9.2	28.7 ± 9.8	29.1 ± 11.9	29.5 ± 11.9	28.7 ± 6.4	28.1 ± 7.7

*BMI* body mass index, *HDL* high-density lipoprotein, *LDL* low-density lipoprotein, *GOT (AST)* glutamic oxaloacetic transaminase/aspartate aminotransferase, *GPT (ALT)* glutamate-pyruvate transaminase 1/alanine aminotransferase, *GTP (γ-GT)* gamma-glutamyl transpeptidase

*p* < 0.05: ^a^vs. CTR; ^b^vs. CTR F; ^c^vs. ALS F; ^d^vs. CTR M

**Table 2:** Characteristics of ALS patients divided by gender

	ALS F (27)	ALS M (25)
Hypertension (Y/N)	7/20	6/19
Dementia (Y/N)	4/23	0/25
ALS-FRS-R score	38.6 ± 6.9	38.4 ± 8.7
Disease duration (months)	25.5 ± 23.2	29.1 ± 32.1
I MN involvement (Y/N)	26/1	22/3
II MN involvement (Y/N)	27/0	25/0
Onset (bulbar/spinal)	7/20	3/22
Bulbar involvement (Y/N)	18/9	10/15
sALS/fALS sera	21 (40.4%)/6 (11.5%)	21 (40.4%)/4 (7.7%)
sALS/fALS CSF	7 (30.4%)/5 (21.8%)	9 (39.1%)/2 (8.7%)

*F* females, *M* males, *MN* motor neuron, *sALS* sporadic ALS, *fALS* familiar ALS

### Obese patients (technical controls)

Few obese subjects (8: 6 females and 2 males) were used as technical controls since serum APN levels are decreased in individuals with visceral obesity [28].

### Other neurological diseases

Forty-seven subjects with different neurological disorders were enrolled (see details in Table 3): 7 FTD, 21 dysimmuno-neuropathy (DN) and 9 AD patients. Additionally, 10 patients with neither the cited neurodegenerative nor inflammatory but other neurological diseases (OND, such as corticobasal degeneration, polyneuropathy, stroke) were included. Duration of symptoms and the primary diagnosis for each disease were based on the corresponding diagnostic criteria [29–31]. Exclusion criteria included patients who did not give consent as well as those affected by metabolic diseases (i.e. metabolic syndrome), glucose intolerance and/or known diabetes.

**Table 3:** Characteristics of patients with neurological diseases

	CTR	ALS	FTD	DN	AD	OND
N	36	52	7	21	9	10
F/M	17/19	27/25	2/5	14/7	4/5	4/6
Age (years)	52.8 ± 17.8	57.9 ± 14.10^c^	73 ± 4.2^a^	50.8 ± 16.8^c,d^	69.9 ± 7.8^a,b^	59.7 ± 20.4
BMI (Kg/m^2^)	22.5 ± 2.5	23.6 ± 3.4^e^	23.2 ± 2.3	22.8 ± 2.5	24.5 ± 3.7	25.3 ± 4.3^a^
Fasting Glucose (mg/dl)	88.1 ± 10.9	90.9 ± 13.8	92.3 ± 12.9	107.1 ± 41.3	99.7 ± 19.6	101.8 ± 17.8
Total Cholesterol (mg/dl)	192.2 ± 25.5	210 ± 44.6	182.2 ± 39.9	217.1 ± 41.4^a^	189.8 ± 32.4	202.5 ± 43.8
HDL (mg/dl)	56.9 ± 17.8	60.4 ± 17.4	49.7 ± 16.5	62.8 ± 16	59.2 ± 15.3	57.8 ± 15.7
LDL (mg/dl)	117.2 ± 39	137.1 ± 37	121 ± 29.1	134.9 ± 41.7	114.4 ± 28	143.6 ± 43
Triglycerides (mg/dl)	94.2 ± 25.3	103.1 ± 29.6	109.5 ± 51.5	111.5 ± 54.9	89.7 ± 30.1	95.4 ± 33.9
GOT (U/L)	18.2 ± 5.6	25.6 ± 10.6^a^	22.5 ± 11.6	18.2 ± 4.8^b^	20.4 ± 4.6	19.6 ± 8.3
GPT (U/L)	18.2 ± 9.3	29.90 ± 16.89^a^	15.7 ± 5^b^	19.6 ± 8.3^b^	19.9 ± 6.2	26.5 ± 18.9
GTP (U/L)	17 ± 6.5	25.8 ± 21.3	16.7 ± 6.7	22.9 ± 11.7	26.1 ± 22.9	34.5 ± 22.6^a^
White Blood Cells (n)	6.9 ± 2.2	6.7 ± 1.7^e^	7.5 ± 1.9	7.3 ± 2.3	6.8 ± 1.8	8.6 ± 2^a^
Lymphocytes (n)	28.9 ± 9.2	28.7 ± 9.8	26.6 ± 6.8	28.3 ± 12.3	30.1 ± 8.7	29.2 ± 9.7

*F* females, *M* males, *BMI* Body Mass Index, *HDL* High density lipoprotein cholesterol, *LDL* Low-density lipoprotein cholesterol, *GOT* (*AST*) Glutamic Oxaloacetic Transaminase/Aspartate aminotransferase, *GPT* (*ALT*) Glutamate-Pyruvate Transaminase 1/Alanine aminotransferase, *GTP* (*γGT*) Gamma-glutamyl TransPeptidase *p* < 0.05 a *vs*. CTR; b *vs*. ALS; c *vs*. FTD; d *vs*. AD; e *vs*. OND

### Collection of biological specimens

#### Blood samples

Two venous blood samples were collected the morning after an overnight fasting from each subject. After blood clot at room temperature, the samples were immediately centrifuged at 2000×*g* for 15 min at +4 °C to obtain serum, aliquoted in vials and stored at −80 °C until use. On the day of analysis, the samples were brought to room temperature and vortexed before analysis.

#### CSF samples

0.5 ml of CSF was collected, under sterile conditions by spinal tap (lumbar puncture), immediately aliquoted, frozen and conserved at −80 °C until use. Blood-contaminated CSFs were excluded from the analysis.

### Sample analysis

#### APN detection

Human APN total levels (all subforms) were measured using a commercially available enzyme-linked immunosorbent assay kit (EIA-4177 DRG® Adiponectin Human ELISA, Springfield, NJ, USA), following the manufacturer’s instructions. Although peripherally the high molecular weight APN is considered the most biologically active, we preferred to evaluate total APN values in both the serum and CNS in order to detect any eventual alteration, as reported in other studies [6]. Sera were diluted 1:500 whereas CSF samples were undiluted. The absorbance measurement at 450 nm was performed using a microplate absorbance reader (Sunrise, Tecan, Männedorf, Switzerland) and data analysed with the provided Magellan™ Data Analysis software.

#### Blood analysis

Standard complete blood screenings were conducted for each patient at the diagnostic unit of our institute certified by the Italian Ministry of Health (see details in Tables 1, 2 and 3).

#### Statistics

The statistical analysis of data was carried out using JMP software, version 3.2.6, SAS Institute Inc, Cary, NC, USA. Means were compared using the non-parametric Wilcoxon test. Pearson’s or Spearman’s rho was used to assess correlations between two variables at a time. A *p* value of 0.05 was the set limit for statistical significance. All the opportune statistical corrections were applied whenever necessary in order to detect unbiased differences among groups.

## Results

### Characteristics of ALS patients vs. CTR

The whole group of ALS patients was comparable to CTR for all the considered parameters, except for glutamic oxaloacetic transaminase/aspartate aminotransferase (GOT (AST)) and glutamate-pyruvate transaminase 1/alanine aminotransferase (GPT (ALT)) levels, which were significantly increased (*p* < 0.05, Table 1). The comparison between ALS and CTR by gender revealed significant variations (Table 1). ALS males were characterized by significantly different values for ALT and AST in comparison to CTR, as for the whole group, whereas in female ALS, only total cholesterol levels were slightly increased. Comparison between ALS females vs. males enlightened significantly higher values in males for enzyme markers of liver disease (GOT (AST) *p* ≤ 0.035; GPT (ALT) *p* ≤ 0.014; GTP (γ-GT) *p* ≤ 0.038). High-density lipoprotein (HDL) was significantly higher only in female ALS, as expected (Table 1).

Although the reported mean disease duration for ALS patients is 25.5 ± 23.2 and 29.1 ± 32.1 months for females and males, respectively (Table 2), the higher values observed for disease duration were mainly due to 3 patients characterized by very slow progression (≥100 months, ≥8 years) jointed to preservation of normal BMI. Hypertension was observed in 25% of ALS patients. No statistically significant differences for ALS Functional Rating Scale-Revised (ALS-FRS-R, see details at https://www.encals.eu/wp-content/uploads/2016/09/ENCALS-SOP-for-ALSFRS-R-v1.2.pdf) and disease duration were observed in ALS patients by sex (Table 2). No statistically different values were observed for blood screenings between fALS and sALS patients (not shown). Finally, almost all ALS female patients were in menopause status.

### APN detection in ALS vs. CTR samples

**Fig. 1:**
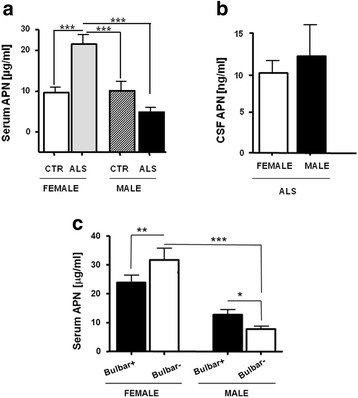
Gender serum APN levels (μg/ml) in the comparison between ALS and CTR (**a**) and CSF APN levels (ng/ml) in ALS patients (**b**). Differences in serum APN between ALS subgroups by gender and bulbar involvement (**c**). Mean ± SE is reported. *p < 0.05, **p < 0.01, ***p < 0.001

We confirmed lower serum APN mean values in OB subjects in respect to CTR (used as technical controls, not shown) [3, 28]. In the whole ALS patient group, serum APN levels were similar when compared to all CTR (18.5 ± 12.3 vs. 14.8 ± 7.8 μg/ml, *p* = 0.33, ns). Nevertheless, gender analysis of serum APN revealed a significant increase only in ALS female patients in respect to both female and male CTR as well as to ALS males (Fig. 1a). Circulating APN levels were higher in ALS females (26.51 ± 11.6 μg/ml) and lower in ALS males (9.8 ± 5.2 μg/ml) than the corresponding CTR (Fig. 1a) whereas comparable amounts were retrieved in CSF (Fig. 1b) between ALS males and females (respectively, 10.43 ± 5.2 vs. 13.5 ± 11.1 ng/ml, *p* = 0.7, ns). Although our ALS cohort comprised 4 females diagnosed with dementia, their exclusion maintained a statistical significance for APN values (*p* ≤ 0.0001, not shown).

No significant APN levels were observed in the CSF of ALS patients by sex (Fig. 1b). Serum APN levels were significantly different when bulbar involvement and gender were considered (see Fig. 1c).

**Fig. 2:**
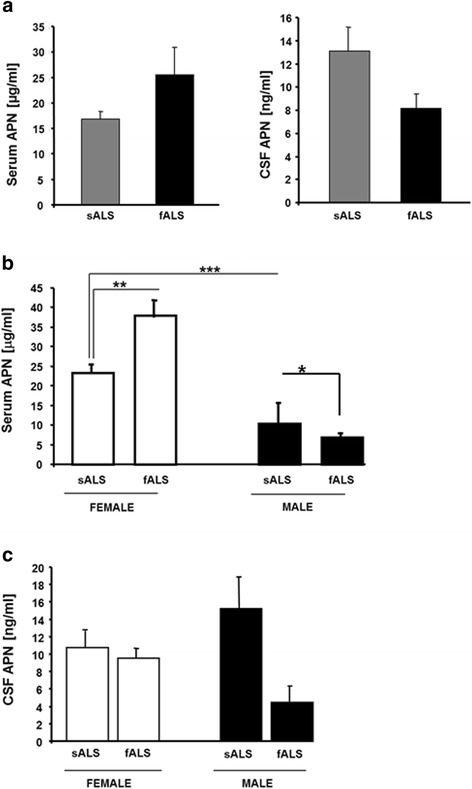
Whole group serum and CSF APN levels in sALS vs. fALS patients (**a**). Comparison of serum (**b**) and CSF (**c**) APN levels in sALS vs. fALS patients stratified by gender. Mean ± SE is reported. *p < 0.05, **p < 0.01, ***p < 0.001

No differential APN levels were retrieved in both serum and CSF, irrespectively of any eventual genetic mutation present in all ALS patients (Fig. 2a). However, the APN levels were significantly higher in female fALS compared to sALS, whereas significant lower levels were observed in fALS males (Fig. 2b). Gender analysis of APN levels in CSF revealed no significant differences between sALS and fALS (Fig. 2c).

### Association with clinical measures

**Fig. 3:**
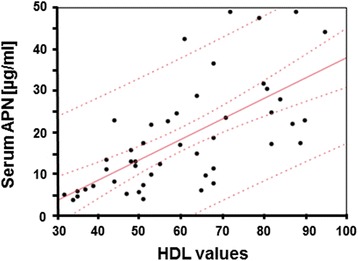
Scatter plot showing the direct correlation between serum APN (μg/ml) and HDL values (mg/dl) within the whole ALS cohort. Number of patients 52, rho = 0.44, ***p < 0.001

Correlation studies between mean serum APN values and clinical parameters of ALS patients enlightened a positive association with HDL (Fig. 3). The significant HDL association with APN was maintained after gender correction (males: rho = 0.56, *p* = 0.003; females: rho = 0.53, *p* = 0.01, not shown). No significant correlations were retrieved for CSF APN levels.

Interestingly, HDL and total cholesterol had an inverse correlation with ALS-FRS-R scores (*p* ≤ 0.01, not shown). Serum APN values in ALS patients analysed by sex showed no significant differences for type of onset, upper or lower motor neuron involvement and disease duration. No associations between bulbar impairment, hypertension or onset and serum APN levels were retrieved.

Only in female ALS, serum APN values had an inverse correlation with ALS-FRS-R scores (rho = 0.24, *p* ≤ 0.004). CSF APN in ALS females was inversely related to the cell number of basophils (rho = −0.63, *p* = 0.011) and monocytes (rho = −0.83, *p* = 0.001). In ALS males, a direct correlation between ALS-FRS-R scores and BMI (rho = 0.48, *p* = 0.04) was found, and we confirmed an inverse one for HDL (rho = −0.53, *p* = 0.01) and total cholesterol (rho = −0.49, *p* = 0.03, not shown) levels.

### APN detection in other neurological diseases

Alterations of serum APN observed in ALS patients prompted us to check this adipokine in other neurological diseases (AD, DN, OND and FTD, which share involvement of the frontal cortex as ALS) in search of specific or common hallmarks within these pathologies.

The clinical characteristics of the studied cohort are detailed in Table 3. No significant differences in the enrolled subjects for BMI were observed since all patients were in the BMI normal range (between 20 and 25 kg/m^2^). The whole ALS cohort was significantly younger than FTD patients (*p* ≤ 0.032) whereas FTD (*p* ≤ 0.014) and AD (*p* ≤ 0.011) patients were significantly older than CTR (Table 3). Significant differences for age were present between DN and FTD (*p* ≤ 0.030) (see Table 3).

**Fig. 4:**
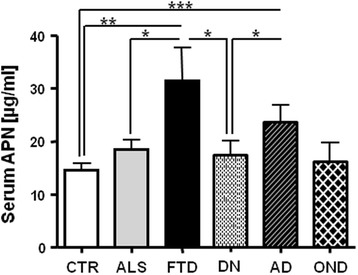
Serum APN levels (μg/ml) in patients affected by different neurological diseases (*ALS* amyotrophic lateral sclerosis, *FTD* fronto-temporal dementia, *DN* dysimmuno-neuropathy, *AD* Alzheimer’s disease, *OND* other neurological diseases) when compared to CTR. Mean ± SE is reported. *p < 0.05, **p < 0.01, ***p < 0.001

The overall analysis of APN levels enlightened that serum APN was significantly increased in AD and FTD patients when compared to CTR (see Fig. 4). Surprisingly, FTD patients displayed the highest serum APN concentration (31.4 ± 15.2 μg/ml), and albeit the restricted number of FTD patients included, their mean APN level was significantly higher than those of DN patients and CTR (Fig. 4). Interestingly, APN levels were also significantly different between ALS and FTD patients, although these pathologies share overlapping features. A significant difference for serum APN level was observed between DN and AD patients (Fig. 4).

Due to the small number of CSF collected for OND, the APN comparison was only restricted to ALS and 3 FTD patients, with a significant difference for high levels in the latter group (10.3 ± 5.7 vs. 20.4 ± 6.3 ng/ml).

## Discussion

We herein demonstrate that serum APN levels characterize ALS patients with normal BMI in a gender-specific manner, and this difference is enhanced in fALS, irrespectively of the genetic mutation carried. The circulating APN amounts also appeared unrelated to CSF levels in ALS patients. In addition, circulating APN levels were altered in different neurological diseases, with the highest levels observed for the very first time in FTD patients.

Interestingly, similar data showing altered expression of several metabolic proteins and adipokines has been previously reported, but only in sALS patients with limb onset [32]. Conversely, our study was focused on early pathophysiological ALS stages when neither metabolic defect- nor systemic inflammatory-altered markers were evident.

In ALS, altered modulation of the APN signalling pathway has been linked to muscle atrophy [12] and energy imbalance has been associated with compromised mitochondrial biogenesis besides oxidative capacity in skeletal muscle [4]. Several mechanisms contribute to altered energy balance in ALS from decreased energy supply and fat storage to increased energy expenditure (reviewed in [18]), but globally, these features appear to affect disease progression more than onset [18]. No influence of riluzole treatment on APN levels has been previously reported [32]. Moreover, in the present study, every ALS patient was treated with riluzole, thus excluding a possible role of this drug on the observed APN levels.

Serum APN levels normally increase linearly with ageing, without any apparent correlation to endogenous sex hormones [33]. A complex interaction between gender and clinical phenotypes has been described in ALS with a higher prevalence of the disease in males, possibly due to the protective effect of oestrogen [34]. Gender is reported to differentially influence muscle fibres, the site of onset, the hormonal asset and the pathological anatomical alterations in ALS [35]. Adiponectin and its receptor R1 regulate a specific disease modifier, peroxisome proliferator-activated receptor gamma coactivator-1α, which is downregulated in ALS human skeletal muscles [4, 36] and is able to modulate disease onset as well as progression in males [37]. The retrieved reduced APN levels in ALS males may therefore be influenced, and possibly explained, also by the gender-specific body fat amount and distribution [38]. Systematic anthropometric studies to determine body composition on a wide cohort of ALS patients are still lacking and deserve investigation.

Independently from the genetic background, altered gender-related APN levels in ALS patients suggest a pleiotropic change in the adipokine network due to the disease affecting several body districts. This anomaly is shared by all ALS patients with normal BMI, worsened in fALS and directly correlated to bulbar involvement. The genetic background of the disease reinforces the gender effect observed for APN levels in ALS.

**Fig. 5:**
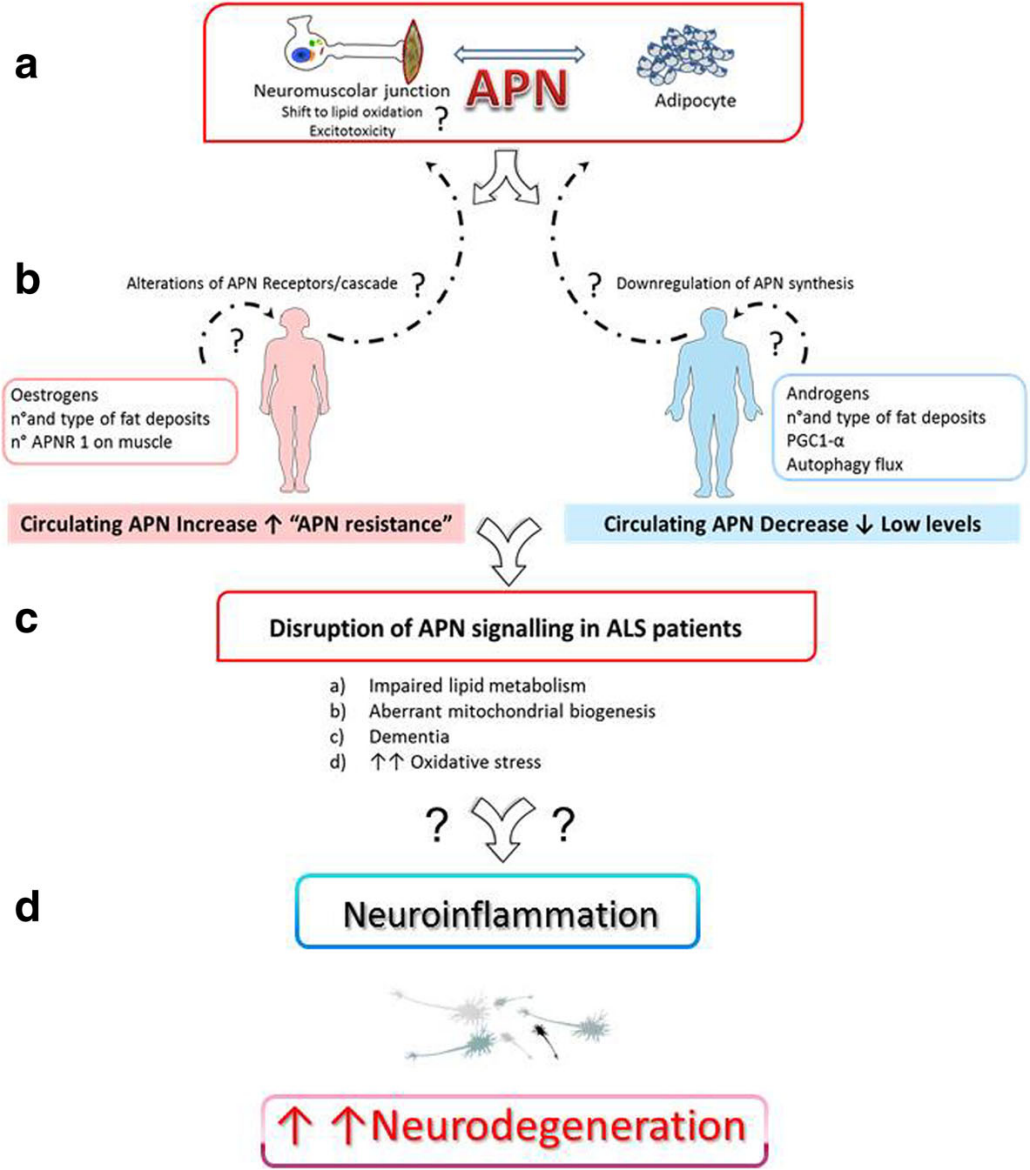
Hypothetical role of gender-specific APN alterations in ALS. Both genders share the same pathological features but opposite serum APN levels (**a**, **b**). Therefore, different biological cascades (**c**) may induce disruption of APN signalling in ALS patients and enhance neuroinflammation and motor neuron degeneration (**d**), so a clear dissection of their single contribution deserves to be deeply investigated

Actually, it is tempting to speculate that gender-specific alterations of circulating APN cause the same biological effect through different mechanisms. Female ALS patients may show a kind of “adiponectin resistance” with desensitized APN receptors, whereas male ALS patients may be characterized by a kind of “adiponectin deficiency” (Fig. 5). Therefore, APN dysregulation may enhance neuroinflammation and exacerbate motor neuron degeneration in addition to ageing, metabolic diseases and viral infections [39]. Concordantly, the neuroprotective effect of a plant APN homolog, osmotin, has already been demonstrated on the lipopolysaccharide (LPS)-induced release of inflammatory mediators, thus reducing the related apoptotic neurodegeneration as well as enhancing synaptic functionality [40].

At the same time, we cannot exclude a gender-different clearance rate of APN at the liver/kidney level [41], as already demonstrated in type 2 diabetes patients wherein APN urinary excretion may be an independent indicator of vascular damage [42].

Interestingly, the direct significant correlation between APN levels and HDL values suggests that this parameter may be predictive of APN quantification in ALS patients. Conversely, APN levels were independent of BMI and systemic inflammatory status in ALS patients, according to previous literature [32].

The association between peripheral and CNS levels is still controversial [3, 43–45], but our data reveal independent amounts within these districts. A similar difference between plasma and CSF was demonstrated for progranulin protein [46], thus suggesting no main alterations of BBB or, alternatively, a compromised modulation of APN in the ALS CNS. The observed APN levels in the CSF of ALS patients are in line with literature data for healthy controls [44].

Adiponectin alterations were observed in all neurological diseases considered, with FTD patients displaying the highest amounts. Although FTD and AD patients were significantly older than the other categories, the appropriate age adjustment maintained the significance, thus suggesting a reduced effect of ageing. In the studied cohort of female ALS, APN levels were extremely elevated and not associated with dementia diagnosis. As already demonstrated in women [47], we cannot exclude that APN increased levels may prospectively act as a “prodromic factor” for dementia/cognitive impairment in ALS. Levels of APN may also be translatable in selective biomarkers for cognitive impairment among two closely related conditions with overlapping clinical features, such as ALS and FTD [13, 22, 23]. However, further investigations are required to better explain the correlation between APN and neuroinflammation.

## Conclusions

Our data demonstrate circulating APN alterations in several neurodegenerative diseases characterized by neuroinflammation. We report a gender-related opposite APN trend in ALS patient sera, but normal levels in their CSF. In addition, in FTD patients sharing some overlapping clinical features with ALS, the highest serum APN levels were observed.

## Abbreviations

AD: Alzheimer’s disease
ALS: Amyotrophic lateral sclerosis
ALT: glutamate-pyruvate transaminase 1/alanine aminotransferase
APN: Adiponectin
AST: glutamic oxaloacetic transaminase/aspartate aminotransferase
CSF: Cerebrospinal fluid
DN: Dysimmuno-neuropathy
FTD: Fronto-temporal dementia
OB: Obese
OND: Other neurological diseases
